# Kinetics modeling, thermodynamics and thermal performance assessments of pyrolytic decomposition of *Moringa oleifera* husk and *Delonix regia* pod

**DOI:** 10.1038/s41598-021-93407-1

**Published:** 2021-07-05

**Authors:** Ayokunle O. Balogun, Adekunle A. Adeleke, Peter P. Ikubanni, Samuel O. Adegoke, Abdulbaset M. Alayat, Armando G. McDonald

**Affiliations:** 1 Department of Mechanical Engineering, College of Engineering, Landmark University, Omu-Aran, Nigeria; 2 Department of Petroleum Engineering, Faculty of Engineering and Technology, University of Ibadan, Ibadan, Nigeria; 3 Department of Forest, Rangeland and Fire Science, University of Idaho, Moscow, ID 83844-1132 USA

**Keywords:** Energy infrastructure, Mechanical engineering

## Abstract

A non-isothermal decomposition of *Moringa oleifera* husk and *Delonix regia* seed pod was carried out in an N_2_ pyrolytic condition with the primary objective of undertaking the kinetics modeling, thermodynamics and thermal performance analyses of the identified samples. Three different isoconversional models, namely, differential Friedman, Flynn–Wall–Ozawa, and Starink techniques were utilized for the deduction of the kinetics data. The thermodynamic parameters were deduced from the kinetic data based on a first-order chemical reaction model. In the kinetics study, a strong correlation (R^2^ > 0.9) was observed throughout the conversion range for all the kinetic models. The activation energy profiles showed two distinctive regions. In the first region, the average activation energy values were relatively higher—a typical example is in the Flynn–Wall–Ozawa technique—MH (199 kJ/mol) and RP (194 kJ/mol), while in the second region, MH (292 kJ/mol) and RP (234 kJ/mol). It was also demonstrated that the thermal process for the samples experienced endothermic reactions thought the conversion range. In summary, both the kinetic and thermodynamic parameters vary significantly with conversion—underscoring the complexity associated with the thermal conversion of lignocellulosic biomass samples.

## Introduction

Pyrolysis is an important thermochemical process for transforming diverse lignocellulosic biomass feedstock into renewable energy products. Biomass is of particular interest in the bioenergy sector because of its carbon neutrality, which could help mitigate the adverse effects of climate change and global warming, if utilized in a sustainable manner. Aside dependence on the carbon-neutrality of biomass to achieve net-zero emission of CO_2_, negative emission technologies (NETs) that include afforestation and reforestation, bioenergy with carbon capture and storage, direct air capture, enhanced weathering and ocean alkalization, biochar-based carbon sequestration could also be deployed in a bid to stabilize the climate^1^. Specifically, biochar, a stable solid carbon-rich material, often produced from biomass pyrolysis, can be applied as soil amendment to sequester carbon. Smith^2^ estimated the soil carbon sequestration and biochar potential as 0.7 Gt carbon eq/year. He noted that they potentially have minimal impact on land use, water, energy demand and cost, albedo, and nutrients; thus, possessing fewer demerits relative to other NETs. Additionally, in a life cycle assessment study of a large-scale slow-pyrolysis plant, Aziz et al.^3^ opined that slow pyrolysis systems have the capacity to generate energy and ensure negative carbon emission.

Notably, biomass feedstock is often available as residues for which there are significant management challenges, particularly, in the developing nations. It has been estimated that about 82.4 Mt of agricultural residues, and 168 Mt of agricultural residues and waste are generated annually in Canada, and Nigeria, respectively^4,5^. Lignocellulosic biomass is a heterogeneous material consisting mainly of an intricate matrix of hemicellulose, cellulose, and lignin; though in varying proportions, depending on the biomass species, age, and environmental conditions. Whereas hemicellulose and lignin are amorphous in nature, cellulose has been noted for its high crystallinity^6,7^. The recalcitrant nature of biomass has made pyrolysis gain wide acceptability because it is suitable for the production of other value-added products, that are considered renewable. A major disadvantage of biomass pyrolysis is the emission of oxides of nitrogen, NO_x_, and this has been reported to be harmful to human health and the environment. The oxides of nitrogen have been implicated in the incidence of acid rain. Furthermore, of the seven notorious oxides of nitrogen, N_2_O has been identified as an ozone-depleting material. It is a greenhouse gas, which have a global warming potential of about 298 times that of CO_2_. Its half-life is between 100 and150 years in the atmosphere^8^. However, there are several robust techniques for mitigating NO_x_ emissions during the thermal degradation of biomass^8^.

Typically, pyrolysis is conducted in the presence of limited supply of air, and it involves intricate reactions that are influenced by biomass composition, heating rate, and temperature. The three main constituents of biomass exhibit different behavior during pyrolytic thermal degradation. Whereas amorphous hemicellulose, which is highly reactive, decomposes earliest at relatively low temperatures, cellulose is fairly stable, thermally, and thus degrades sharply over a narrow peak range^3^. In the case of lignin, it is the least reactive compound and it degrades over a broad range of temperature; leading to a much less volatilization relative to the other constituents. In terms of the thermal operating conditions, it has been observed that slow heating rates, long residence time and relatively low temperature favor char production^1^. Conversely, bio-oil generation is mostly achieved through rapid heating rates, short vapor residence time and moderate temperature. A major attraction to pyrolysis is the diversity of product it generates, namely, bio-oil, syngas, and biochar. Bio-char can be deployed as solid fuels, activated carbon, and for soil enhancement applications to promote carbon sequestration^8,9^. Syn-gas and bio-oil possess high heating values making them valuable for energy recovery purposes. Furthermore, bio-oil can be further refined into improved quality fuels to replace gasoline, diesel, and chemicals obtained from non-renewable sources.

Aside investigation into the medicinal value of *Moringa oleifera* and the ornamental application of *Delonix regia*, there have been few thermochemical studies on their various parts^10,11^. Titiloye et al.^12^ in a bid to examine the potential of some biomass wastes as feedstock for thermochemical conversion undertook the characterization of *M. oleifera* seed cakes alongside other agricultural materials. They noted that all the samples examined had low nitrogen and sulphur contents. Rashid et al.^13^ investigated *M. oleifera* oil as a possible feedstock for biodiesel production through a standard transesterification procedure and they concluded that the oil could be a suitable feedstock for biodiesel. Researchers have also evaluated the *M*. *peregrina* seed husk and oil cake as biofuel potential sources; conducting detailed thermochemical and physicochemical characterization on these parts^10^. They came to the conclusion that *M*. *peregrina* is a potential source of bio-fuel and food. Regarding *D. regia*, Kawale and Kishore^14^ subjected *D. regia* to pyrolysis in a tubular reactor at different temperatures, and then undertook comprehensive analyses of the solid and liquid products. Their results indicated that *D. regia* represents a suitable feedstock resource for both biofuels and value-added green chemicals. Apparently, some of the investigations concentrated mainly on physico-chemical characterization, while others examined the use of the seed oils and other plant parts aside the *M. oleifera* husk (MH) and the *D. regia* pod (RP). The seeds of MH as well as the oil are edible products. This could lead to the challenge of an unhealthy competition between food and fuel. It is therefore imperative to explore other parts that are less likely to compete with food and avoid raising grave public ethical issues^15^. The focus of the current study is to investigate the kinetics, and thermodynamics analyses of both agricultural residues (MH husk and RP pods) under pyrolytic conditions.

The role of kinetics modeling is vitally critical to understanding the underlying phenomena and mechanisms that govern thermal processes, making it a complementary tool to experimental analysis. It has been noted that pyrolysis kinetics could produce sub-models that could be coupled to transport phenomena equations to aptly describe thermochemical conversion processes, and also help in the design of efficient reactors^4,16,17^. To obtain the kinetic data, either model-fitting or iso-conversional techniques could be utilized. The iso-conversional methods have attracted greater attention because it is not necessary to have a foreknowledge of the decomposition mechanism, which relies more or less on subjective judgment. This obviates the need for the inclusion of a decomposition model in the rate equation and thus, relatively, simplifies the determination of the kinetic parameters. However, it requires multiple TGA measurements at a minimum of four different heating rates^18^. Vyazovkin et al.^19^ recommended that the difference between the lowest and highest heating rate should be at least an order of magnitude—starting with a very low heating rate (e.g., 2.5 °C/min).

Thermodynamic parameters such as changes in enthalpy ($\Delta H$), Gibbs free energy ($\Delta G$), and entropy ($\Delta S$) are valuable in gaining insight into the feasibility of reaction, reactivity, and the direction of energy conversion^20^. These parameters can be quantified from kinetic data. Though, on a relatively smaller scale, a robust tool for the evaluation of the thermal degradation of biomass is the thermogravimetric analysis (TGA). The kinetic and thermodynamic parameters could then be deduced from the TGA data. In the investigation of the kinetics and reaction chemistry of tobacco waste pyrolysis, it was observed that the effective activation energy was strongly dependent on conversion ratio and it was calculated as 144–338 kJ/mol^4^. Müsellim et al.^21^ determined the kinetic and thermodynamic parameters of the pyrolytic decomposition of pea waste biomass. The activation energy, and change in Gibbs free energy were found to be in the range of 184–312 kJ/mol, and 143–148 kJ/mol respectively. Furthermore, Huang et al.^20^ conducted kinetic, thermodynamic, TG and Py-GC/MS analyses on thermal conversion of spent mushroom substrate in CO_2_ and N_2_, and they noted comparatively higher thermodynamic parameters in the N_2_ environment.

The investigations that have been undertaken in pyrolytic studies have focused mostly on the thermal degradation of a single stream of agricultural waste, and even much less have investigated the kinetic and thermodynamic analyses of *M. oleifera* husk (MH) and *D. regia* seed pod (RP). Furthermore, some of the exploration of the *M. oleifera* for bioenergy applications have centered on the edible parts as earlier noted and this represents a potential ethical risk. It is therefore important to examine the non-edible parts of these plants both for waste management and environmental compatibility purposes. To the best of the author’s knowledge, this is perhaps the first attempt to compare these agricultural waste streams in an N_2_ inert atmosphere. Therefore, the aim of this research work was to degrade MH and RP in an N_2_ pyrolytic atmosphere under non-isothermal TGA heating condition. Subsequently, the TGA data will be used to evaluate the kinetic, thermodynamic, thermal performance parameters. The kinetic study was based on iso-conversional techniques.

## Materials and methods

### Materials

The MH and RP samples were collected in Landmark University premises in June 2020. The samples were collected as waste materials, which is totally in accordance with the ethics of waste management in Nigeria. The samples were considered as wastes and are released to researchers to freely turn into wealth with no need for written permission. For ease of grinding, the samples were oven dried (Convection oven) at 70 °C for 24 h, and milled in ball mill. Using a mechanical sieve, the samples were sieved into 0.6- and 1.18-mm particle sizes. The cellulose, xylan from corn, softwood kraft lignin, hardwood organosolv lignin, respectively were procured from Whatman CF1, TCI America, Indulin AT-Mead Westvaco, and Lignovate LLC and they were used as received.

### Physico-chemical and structural characterization of MH and RP

The proximate analysis, and the higher heating value determination [in a Parr oxygen bomb calorimeter (model 1261)] were done according to ASTM E870-82 and ASTM D5865-04, respectively. A Costech ESC 4010 instrument was used for the determination of the elemental analysis to obtain the C and N contents. The extractive, lignin, and carbohydrate contents determination for MH and RP were undertaken according to relevant ASTM standards such as ASTM D 1108-96, ASTM D 1106-96, and ASTM E 1758-01, respectively. Similarly, the X-ray diffractogram and the FTIR spectroscopy analyses as well as the relevant cellulose crystallinity monitoring variables were undertaken^22–24^.

### Non-isothermal thermogravimetry

The feedstock samples (MH and RP) were subjected to dynamic heating measurements in a Perkin Elmer TGA-7 instrument. The heating temperature was ramped up from environmental conditions (28.9 °C) to 900 °C at varied heating rates (5, 10, 20, and 50 °C/min). The carrier gas was N_2_ at a flow rate of 30 mL/min. The data obtained were analyzed using the Pyris v11 software. The ICTAC recommendation on kinetic data gathering and processing were largely followed with at least an order of magnitude difference between the least and the highest heating rate. Furthermore, to ensure reproducibility and accuracy of the TGA data the heating rate at 20 °C/min was repeated^18^.

### Kinetic modelling

The single step global kinetic model is often deployed for describing the solid-state degradation in an isothermal heating condition. The model that captures this thermal process is given as a product of conversion function and Arrhenius relation (Eq. (1)).

1 $$\frac{{d\theta }}{{dt}} = Aexp^{{\left( { - \frac{E}{{RT}}} \right)~}} f\left( \theta \right),$$

where *R* = universal gas constant (8.314 J/mol K), $f\left( \theta \right)~$ = differential decomposition model, and $\theta$ = conversion degree expressed as Eq. (2).

2 $$\theta = \frac{{W - W_{i} }}{{W_{f} - W_{i} }}.~$$

The $$W$$, $$W_{i}$$,$$~W_{f}$$, respectively are sample weight (%) at temperature T, initial weight and residual weight. The relevant non-isothermal heating model is derived from the assumption of constant linear heating rate, $$\beta = {\raise0.7ex\hbox{${dT}$} \!\mathord{\left/ {\vphantom {{dT} {dt}}}\right.\kern-\nulldelimiterspace} \!\lower0.7ex\hbox{${dt}$}}$$, yielding Eq. (3)

3 $$\frac{{d\theta }}{{dT}} = \frac{A}{\beta }exp^{{\left( { - \frac{E}{{RT}}} \right)~}} f\left( \theta \right).~~$$

### Differential Friedman (DFM) method

Taking the natural logarithm of Eq. (3), yields differential Friedman’s kinetic model (Eq. (4)).

4 $$\ln \left[ {\frac{{d\theta }}{{dt}}} \right] = \ln \left[ {\beta \left( {\frac{{d\theta }}{{dT}}} \right)} \right] = \ln \left[ {Af\left( \theta \right)} \right] - \frac{E}{{RT}}.$$

In the DFM the conversion function, $f\left( \theta \right)$, is assumed constant. The implication of this is that while the solid-state degradation is not dependent on the temperature, it is mainly dependent on the rate of mass conversion. The linear plot of $\ln \left[ {\frac{{d\theta }}{{dt}}} \right]$ against $\frac{1}{T}$ is generated for different heating rates and the apparent activation energy is determined from the slope $\left( {slope = - \frac{E}{R}} \right)$. It is important to note that the use of the derivative conversion data makes DFM prone to experimental “noise” and numerical instability, and therefore caution must be exercised in the data interpretation^25^.

### Flynn–Wall–Ozawa (FWO) method

The FWO model assumes a constant apparent activation energy in the course of the thermal decomposition process and approximates the temperature integral function using Doyle’s relation. Applying logarithm to the integral function and inserting Doyle’s approximation yields Eq. (5).

5 $$\log \beta = \log \left( {A\frac{E}{{Rg\left( \theta \right)}}} \right) - 2.315 - 0.4567\frac{E}{{RT}}.$$

A plot of $\log \beta$ against $\frac{1}{T}$ for different heating rates produces straight lines from which the apparent activation energy can be evaluated as $\left( {slope = - ~\;0.4567\frac{E}{R}} \right)$.

### Starink (STK) method

The Starink method optimized two isoconversional methods, namely, FWO and KAS, and came up with Eq. (8) ^21^.

6 $$\ln \left( {\frac{\beta }{{T^{{1.92}} }}} \right) = C_{s} - 1.0008\frac{E}{{RT}}.$$

Again, a plot of $\ln \left( {\frac{\beta }{{T^{{1.8}} }}} \right)$ against the reciprocal of temperature generates linear curves and the apparent activation energy can be computed from their slopes.

### Thermal performance parameters

The thermal parameters, namely, ignition temperature (*T*_*i*_), the temperature at the maximum DTG (*T*_*max*_), the burnout temperature (*T*_*b*_), the corresponding time (*t*_*i*_, *t*_*max*_, *t*_*b*_), the maximum and average DTG (− *R*_*p*_ and − *R*_*v*_) were obtained from TGA measurements^26,27^. These were subsequently used to calculate the performance parameters which include comprehensive flammability (*S*), flammability (*C*), ignition (*D*_*i*_) and burnout (*D*_*b*_) according to the formulas in Eqs. (7)–(10).

7 $$S = \frac{{ - R_{p} \times - R_{v} }}{{T_{i}^{2} \times T_{b} }},$$

8 $$C = \frac{{ - R_{p} }}{{T_{i}^{2} }},$$

9 $$D_{i} = \frac{{ - R_{p} }}{{t_{i} \times t_{b} }},$$

10 $$D_{b} = \frac{{ - R_{p} }}{{\Delta t_{{1/2}} \times t_{p} \times t_{b} }}.$$

### Thermodynamic analysis

The kinetic data were deployed for the computation of the thermodynamic parameters [changes in enthalpy $\left( {\Delta H,~{\text{J}}/{\text{mol}}} \right)$, Gibbs free energy $\left( {\Delta G,~\;{\text{J}}/{\text{mol}}} \right)$, and entropy $\left( {\Delta S,~\;{\text{J}}/{\text{mol}}} \right)]$ as based on Eqs. (11)–(13).

11 $$\Delta H = E_{\theta } - RT,$$

12 $$\Delta G = ~E_{\theta } + RT_{{max}} \ln \left( {\frac{{k_{B} T_{{max}} }}{{hA_{\theta } }}} \right),$$

13 $$\Delta S = \frac{{\Delta H - \Delta G}}{{T_{{max}} }},$$

where $k_{B}$, and $h$ are the Boltzmann constant $\left( {1.381 \times 10^{{ - 23}} {\text{J}}/{\text{K}}} \right)$ and Planck constant $\left( {6.626 \times 10^{{ - 34}}\, {\text{J s}}} \right)$, respectively.

## Results and discussion

### Basic characterization, FTIR, and XRD analyses of MH and RP

**Figure 1:**
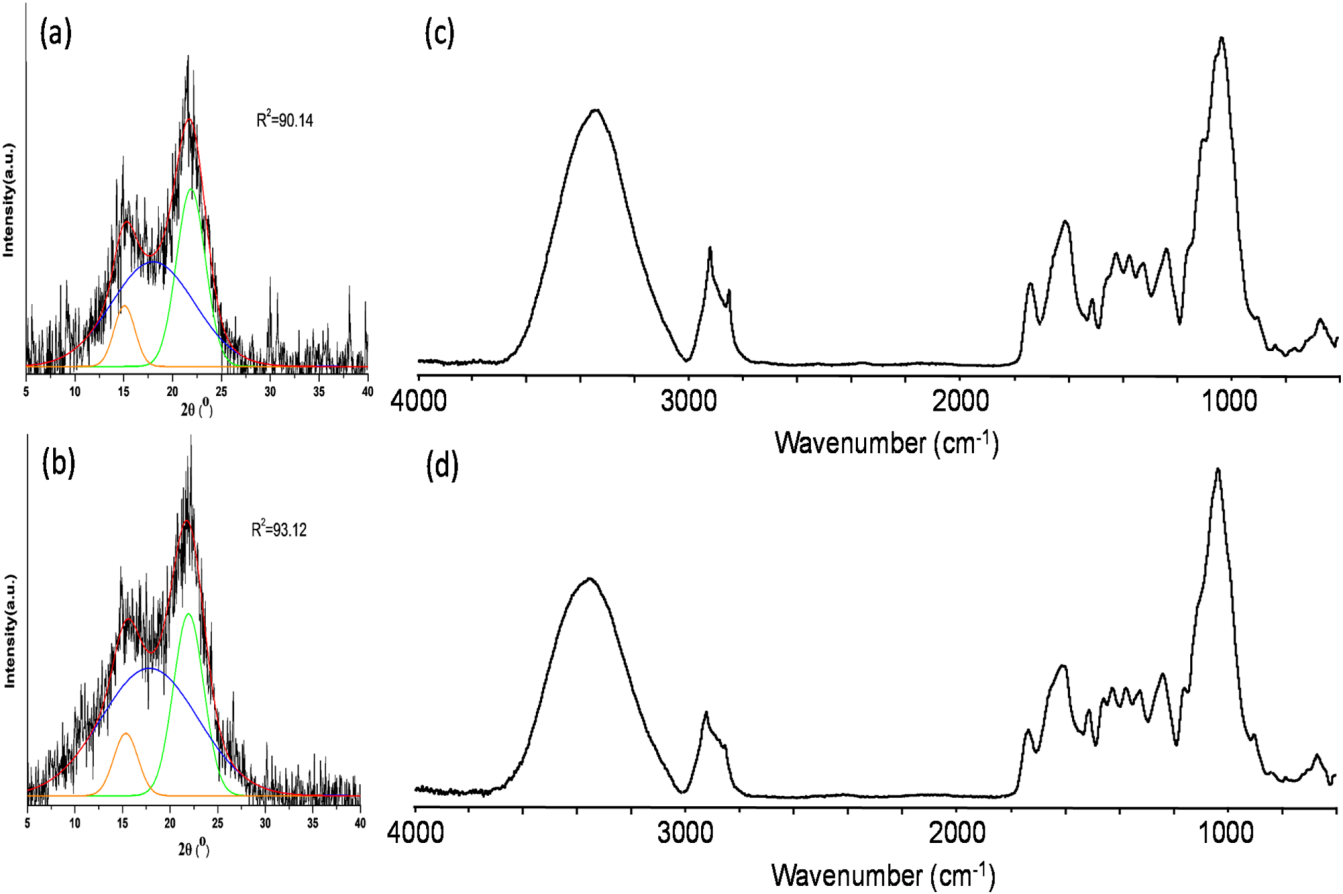
X-ray diffractograms of (**a**) *M. oleifera* husks (MH), (**b**) *D. regia* seed pod (RP) and FTIR spectra of (**c**) MH and (**d**) RP.

Table 1 shows the results of the proximate, compositional (ash, extractives, lignin and carbohydrate), elemental, and HHV analyses of MH and RP. Aside the CH_2_Cl_2_ extractives, the data for other parameters are comparably close. Figure 1a–d, respectively present the data for XRD (MH and RP) and FTIR (MH and RP). The characterization data presented here have been extensively discussed in another study, which is under-review^28^.

**Table 1:** Proximate, elemental, and compositional data for MH and RP sample.

Parameter	MH	RP
Volatile matter (VM) (%)	81.3 ± 0.2	79.5 ± 0.4
Fixed carbon (FC) (%)	9.93 ± 0.55	13.4 ± 0.6
Ash (%)	4.35 ± 0.05	4.82 ± 0.11
Higher heating value (HHV) (MJ kg^−1^)	16.6 ± 0.2	15.9 ± 0.1
CH_2_Cl_2_ extractives (%)	3.35 ± 0.02	0.53 ± 0.02
Acid soluble lignin (%)	3.64 ± 0.04	1.87 ± 0.01
Klason lignin (%)	17.9 ± 0.1	22.2 ± 0.2
Total lignin (%)	21.5 ± 0.1	24.1 ± 0.2
C (%)	44.9	47.3
N (%)	1.7	1.0
Protein (N × 6.25) (%)	10.6	6.3
Glucan	31.4 ± 0.5	41.1 ± 0.5
Xylan	18.5 ± 0.5	17.9 ± 0.5
Galactan	3.70 ± 0.05	1.03 ± 0.05
Arabinan	1.50 ± 0.05	0.40 ± 0.02
Mannan	0.30 ± 0.02	0.20 ± 0.02
Total neutral sugar (%)	55.4 ± 1.1	60.6 ± 1.1

### Thermogravimetric experiments of MH and RP in N_2_ pyrolytic environment

**Figure 2:**
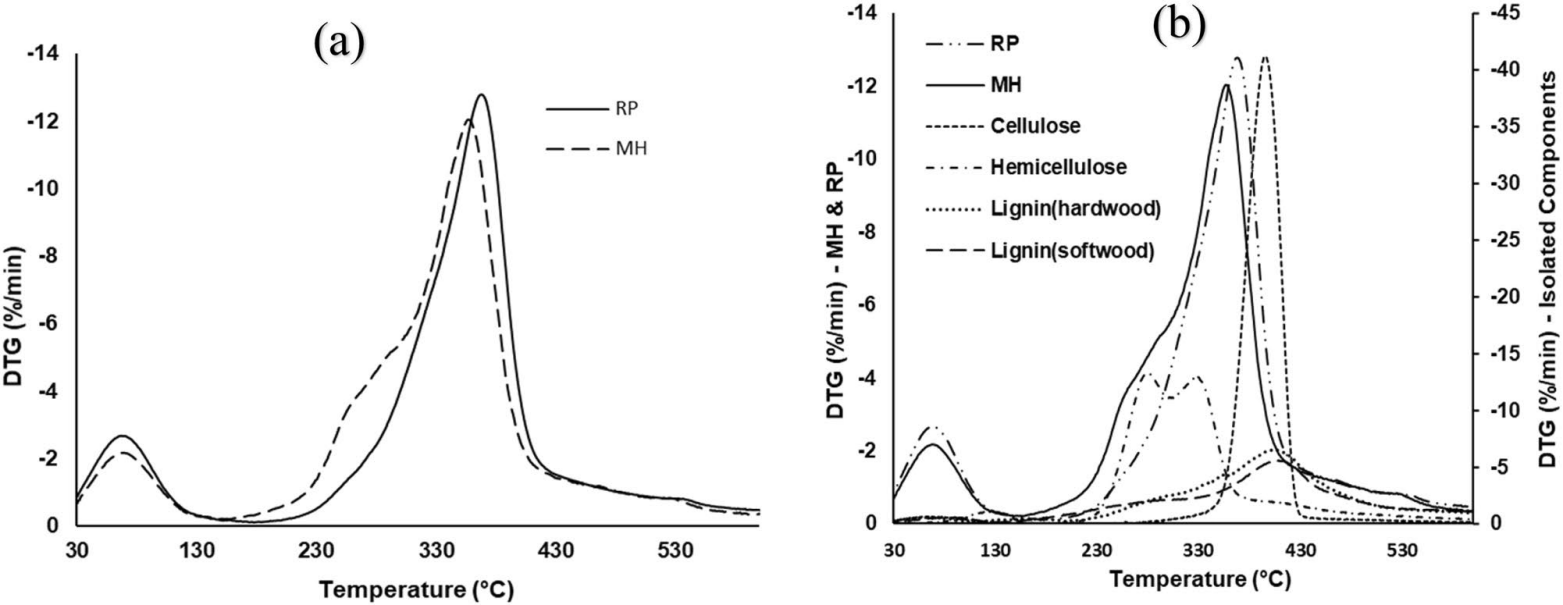
DTG thermograms for (**a**) MH and RP (**b**) with isolated constituents of lignocellulosic biomass in N_2_ pyrolytic atmosphere at 20 °C/min.

Figure 2a shows the DTG thermograms for the pyrolytic degradation of MH and RP in N_2_ at 20 °C/min, while Fig. 1b includes those of the isolated constituents of lignocellulosic biomass sample. The decomposition profiles for MH and RP are fairly similar with the emergence of visible DTG peaks around 70 and 370 °C, and characteristic “shoulders” just before the main DTG peaks at about 250 °C. The first set of peaks signifies water evaporation, and it corresponds to about 5% weight loss. The highest DTG peaks for MH and RP at about 370 °C are indicative of cellulose degradation. It has been opined that cellulose is completely degraded at temperatures above 250 °C^29^. This is lucidly attested to in Fig. 2b with the appearance of a sharp peak at about 400 °C for cellulose. The apparent difference in the height of the cellulose peak (− 40%/min) relative to the main DTG peaks in MH (− 12%/min) and RP (− 13%/min) indicates a higher rate of decomposition. This may be due to the fact that the isolated cellulose is devoid of the limitations of the complex bonds associated with the macromolecular polymer structure of biomass. The appearance of the “shoulder” is typically assigned to hemicellulose decomposition, and the MH curve shows a more notable one. This implies that in comparison to RP, at about 260 °C more of MH is degraded. A similar observation has been made in literature, though, with respect to hardwood and softwood^6^. It is also significant to note that xylan, which is representative of an angiosperm hemicellulose, decomposed at about this temperature—confirming the hemicellulose decomposition hypothesis at this point. The imperceptible peaks at around 600 °C may suggest the disassociation of the strong bonds in lignin and/or thermal breakdown of some residual compounds derived from the previous primary reactions^22,30^.

**Figure 3:**
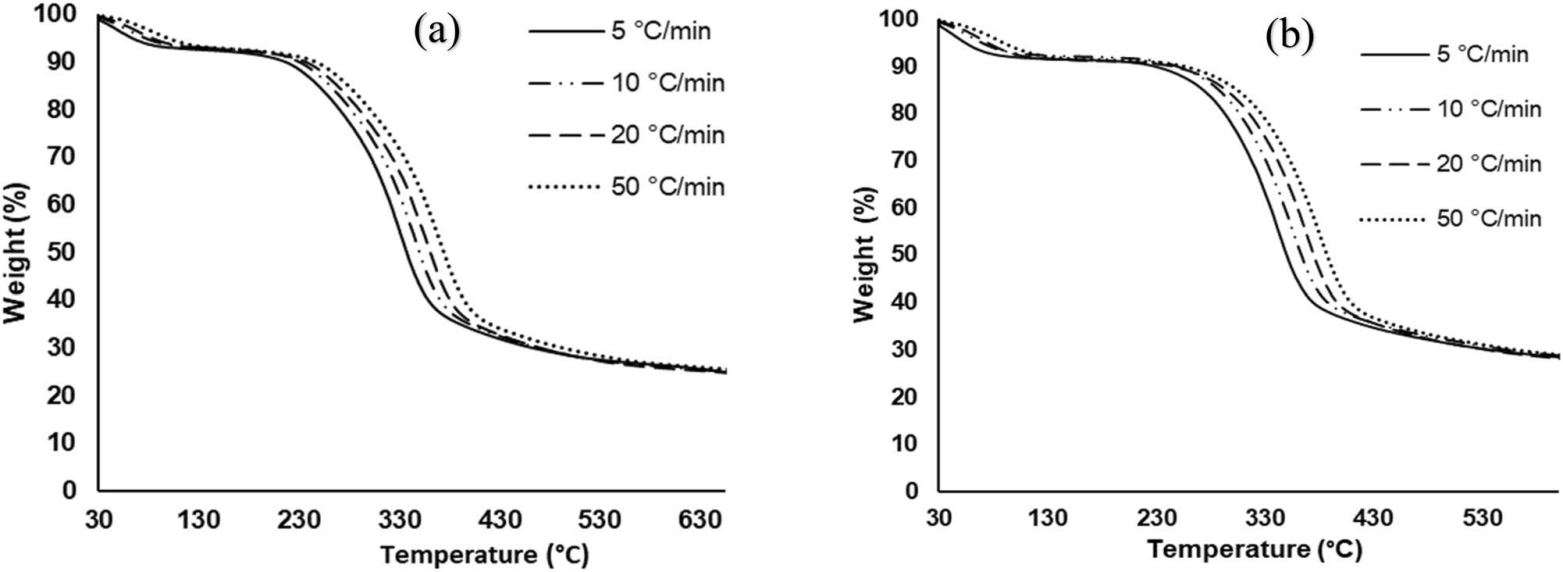
TGA thermograms at 5, 10, 20, 50 °C/min for (**a**) MH and (**b**) RP in N_2_ pyrolytic environment.

Figure 3 depicts the TGA thermograms for the thermal decomposition of MH and RP in N_2_ pyrolytic atmosphere, while Table 2 presents some characteristic peaks in the DTG thermograms for both samples at multiple heating rates. Generally, the curves shift to higher temperatures with an increase in heating rates. Similarly, an increase in heating rates led to a rise in the height of the DTG curves. For example, the maximum peaks in absolute terms for RP at 5, 10, 20, 50 K/min reads 3.23%/min (342 °C), 6.44%/min (356 °C), 12.8%/min (368 °C), and 30.6%/min (380 °C) respectively. An increase in the heating rate leads to rapid heating at the external parts relative to the interior of the sample, thus, pushing the degradation temperatures to higher values. These trends have been reported in literature^31,32^. A marginal variation was noted in the residual weight for both samples. This may be an attestation to the relatively close compositional makeup, as shown in Table 1. In addition, a negligible variation in the residual weight for each sample was observed with successive increase in the heating rate. This implies that given the prevailing thermal conditions, the decomposition of the feedstock sample is not dependent on the heating rate.

**Table 2:** Heating rate, and DTG peak temperatures of MH and RP decomposition in N_2_ pyrolytic environment.

$\beta$ (°C/min)	MH			RP		
	$T_{{max}}$ (°C)	DTG (%/min)	$R_{w}$ (%)	$T_{{max}}$ (°C)	DTG (%/min)	$R_{w}$ (%)
5	42.3	− 0.65	18.2	49.5	− 0.82	23.2
	328.1	− 3.03		342.4	− 3.23	
	642.5	− 0.15		637.1	− 0.14	
10	59.6	− 1.14	20.2	60.6	− 1.34	24.1
	345.0	− 6.04		355.8	− 6.44	
	668.3	− 0.26		660.1	− 0.26	
20	71.9	− 2.16	20.3	67.5	− 2.68	23.9
	357.1	− 12.0		367.9	− 12.8	
	690.8	− 0.49		672.9	− 0.47	
50	83.7	− 4.78	21.2	82.9	− 5.64	24.6
	368.8	− 28.5		379.7	− 30.6	
	716.9	− 1.20		699.6	− 1.14	
Average			20.0			24.0

$\beta$ heating rate, $T_{{max}}$ maximum peak temperature, $R_{w}$ residual weight.

### Kinetic modeling

#### Model-free technique

**Figure 4:**
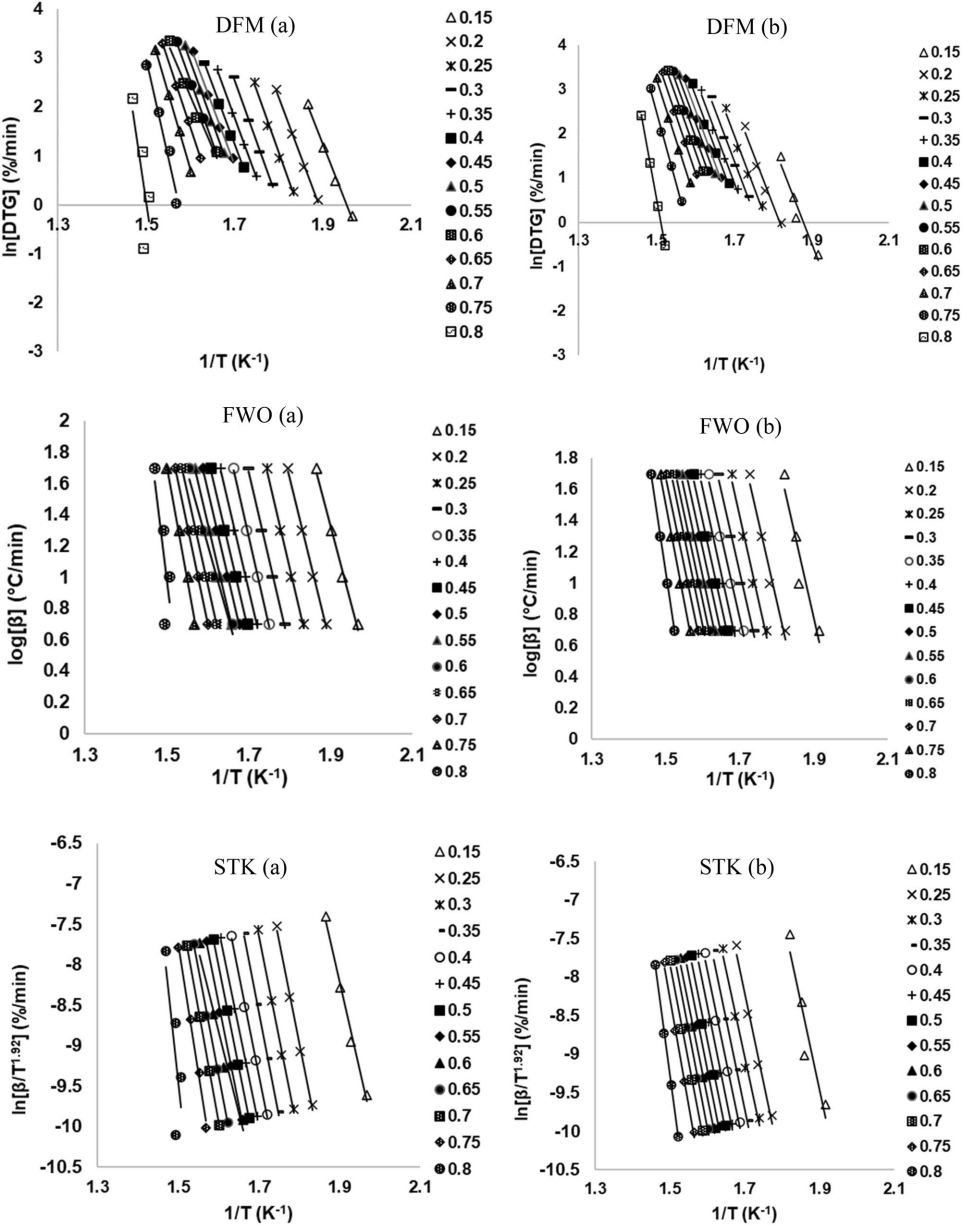
Graphs of iso-conversional lines of DFM, FWO, STK models for (**a**) MH and (**b**) RP in N_2_ inert environment.

Figure 4a,b show the iso-conversional plots for MH and RP samples as obtained from the application of Eqs. (4)–(6). The limit of conversion, $\theta$, was restricted to 0.15–0.80 with an increment of 0.05. In this conversion range, a strong correlation (R^2^ > 0.9) was exhibited for the three models, thus, representing a relatively high degree of accuracy. This also underscores an important point—that the values of E_θ_ are not functions of the heating rates and, therefore they may be considered to be the effective activation energies^9^. Furthermore, outside this limit, poor correlations were exhibited. This may be an indication of the inability of chemical kinetics model to aptly capture the type of reaction involved at the early stages of decomposition. Those stages are typically characterized by the release of both physically and chemically bound water molecules, as well as some low organic volatiles.

**Figure 5:**
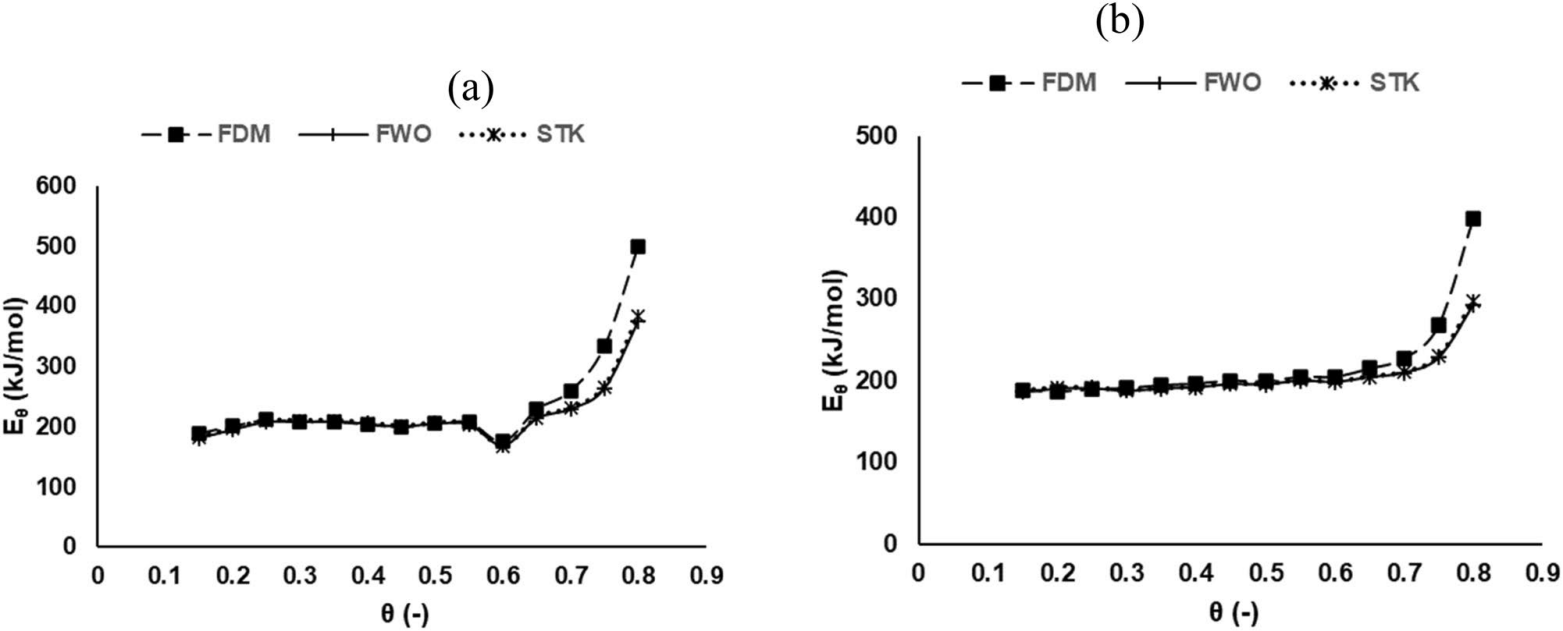
Plots of apparent activation energy, *E*_θ_, against conversion ratio, θ, (**a**) MH and (**b**) RP in a pyrolytic environment.

The dependence of E_θ_ on conversion is clearly demonstrated in Fig. 5a,b. Notably, the trajectory of the E_θ_ curve for each sample are quite similar, showing two distinctive regions. A similar result has been reported in published literature^6^. The first region, 0.15 $\le \theta \le$ 0.60, for DFM, FWO and STK respectively, had average values of E_θ_ for MH (201, 199 and 200 kJ/mol) and for RP (196, 194 and 194 kJ/mol). In this region, devolatilization is the predominant reaction that occurs leading to a simultaneous breakdown of hemicellulose, cellulose, and lignin; albeit in varying degrees^9^. The other region, 0.65 $\le \theta \le$ 0.80, had average values of E_θ_ that were relatively higher than the first region—MH (364, 292 and 296 kJ/mol) and for RP (277, 234 and 236 kJ/mol). It has been opined that this region witnesses mostly lignin degradation and char formation^4^. The E_θ_ values obtained in this study compare well with other studies^25^. The minimum energy required to be surmounted for a reaction to take place is referred to as the activation energy, which is dependent on the related decomposition mechanisms. By implication, a high E_θ_ makes the initiation of a reaction comparatively more difficult. Thus, the initiation of the decomposition processes for MH and RP at the early stages was relatively easier as indicated by values of lower energy barrier. Generally, the average value of E_θ_ obtained for DFM is slightly higher than the other two methods. The DFM is a differential method that utilizes the derivative conversion data; making it susceptible to experimental “noise” and numerical instability^25^. This notwithstanding, the result obtained is still relatively more accurate. On the other hand, the average values derived from FWO and STK exhibited negligible variations. Apparently, both techniques are premised on an oversimplified temperature integral approximation. The apparent variation in E_θ_ with conversion highlights the intricate reaction mechanisms and pathways associated with the thermal conversion of MH and RP samples. The thermal decomposition of biomass comprises of diverse reaction mechanisms that may include diffusion, nucleation, and interface occurring concurrently and/or successively.

### Thermal performance parameters of MH and RP

The thermal performance parameters for MH and RP under pyrolytic decomposition is shown in Table 3. They were obtained from the thermogravimetric measurements data and Eqs. (7)–(10). The performance parameters, *S*, *C*, *D*_*i*_ and *D*_*b*_, all increased with increasing heating rates. It is noteworthy that the increase was fairly of the same order of magnitude for both samples. High values of *D*_*i*_ are indications of better ignition capability. Again, a rise in heating rates led to a corresponding increase in the ignition, peak, and burnout temperatures with a corresponding decrease in time. This trend agrees with data in published literature^27^.

**Table 3:** Pyrolytic thermal performance of MH and RP at different heating rates.

MH													
β (K/min)	*T*_*i*_ (K)	*T*_*max*_ (K)	*T*_*b*_ (K)	*t*_*i*_ (min)	*t*_*p*_ (min)	*t*_*b*_ (min)	$\Delta t_{{1/2}}$(min)	* − R*_*p*_ (%/min)	* − R*_*v*_ (%/min)	*S* (%^2^/(min^2^ K^3^))	*C* (%/(min K^2^))	*D*_*i*_ (%/min^3^)	*D*_*b*_ (%/min^4^)
50	571.4	642.0	1008.9	5.7	7.2	14.02	7.8	28.5	4.52	3.91E−07	8.73E−05	6.93E−01	3.64E−02
20	560.9	630.3	760.0	13.4	17.0	23.9	18.4	12	1.83	9.17E−08	3.81E−05	5.25E−02	1.61E−03
10	550.0	618.2	668.5	25.5	32.7	38.05	35.4	6.04	0.92	2.74E−08	2.00E−05	7.24E−03	1.37E−04
5	533.1	601.3	638.4	47.9	62.2	70	68.4	3.03	0.47	7.85E−09	1.07E−05	1.02E−03	1.02E−05
RP													
50	593.9	652.9	994.2	6.22	7.43	13.8	10.3	30.6	4.33	3.78E−07	8.67E−05	6.62E−01	2.91E−02
20	583.5	541.1	755.3	14.6	17.6	23.7	24.9	12.8	1.75	8.70E−08	3.76E−05	5.00E−02	1.24E−03
10	558.3	629.0	673.6	26.4	33.9	38.7	47.0	6.44	0.872	2.67E−08	2.07E−05	7.20E−03	1.04E−04
5	553.2	615.6	646.5	51.8	65.0	71.5	92.6	3.23	0.441	7.20E−09	1.06E−05	9.59E−04	7.51E−06

### Thermodynamics analysis

**Figure 6:**
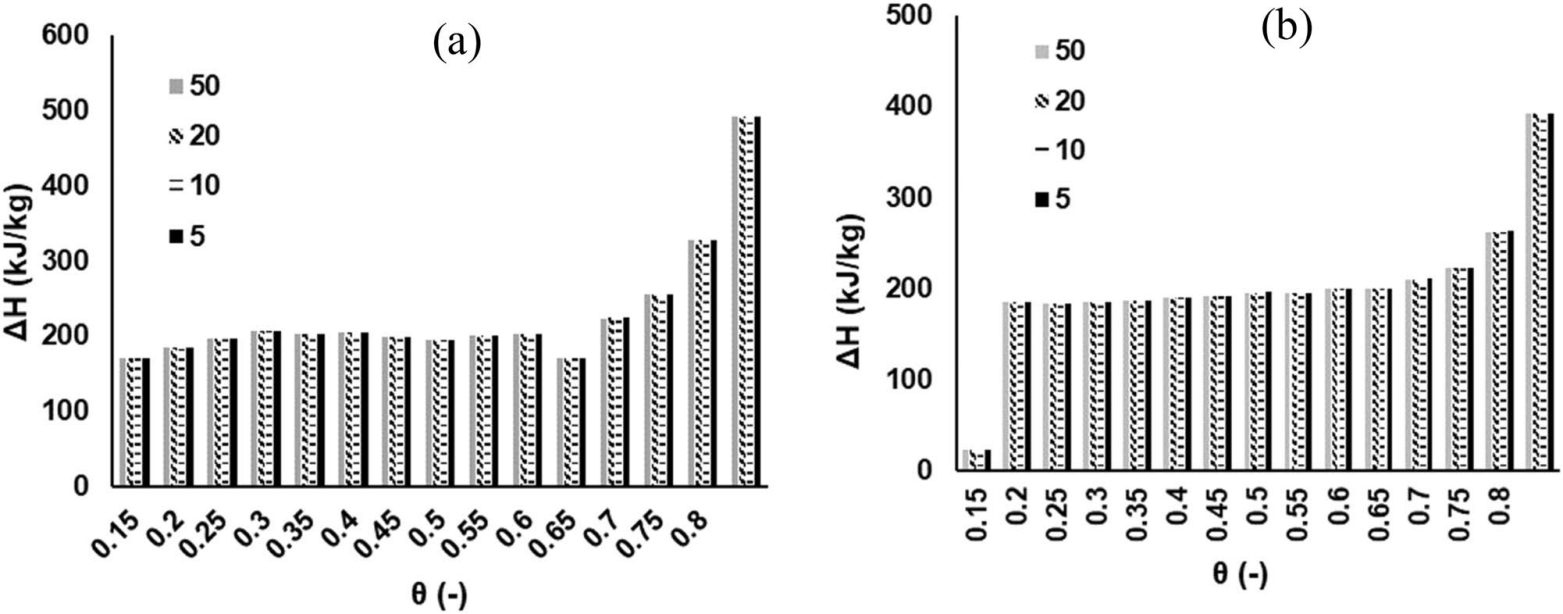
Plot of change of enthalpy ($\Delta H$) at 5, 10, 20, 50 K/min based on DFM (N_2_ pyrolytic environment) for (**a**) MH and (**b**) RP thermal decomposition.

**Figure 7:**
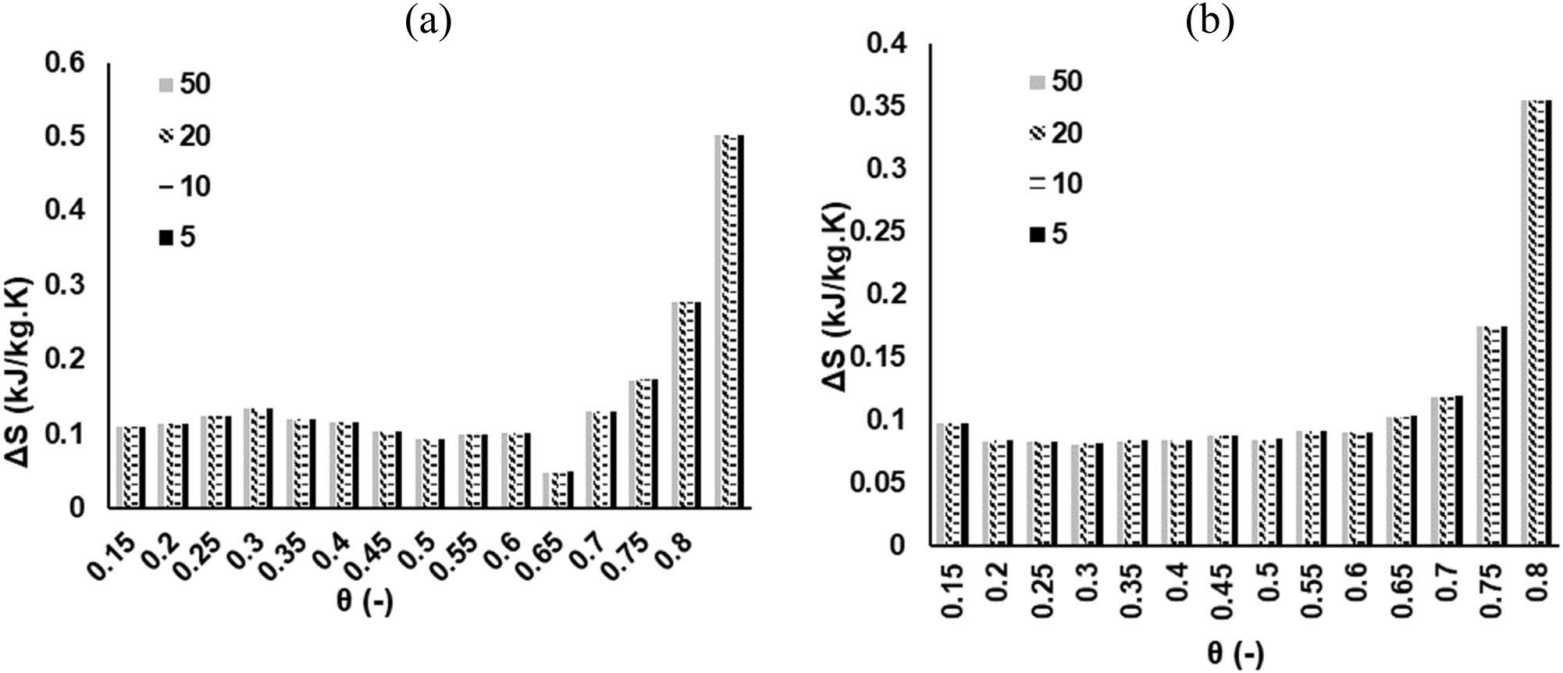
Plot of change of entropy ($~\Delta S$) at 5, 10, 20, 50 K/min based on DFM (N_2_ pyrolytic environment) for (**a**) MH and (**b**) RP thermal decomposition.

**Figure 8:**
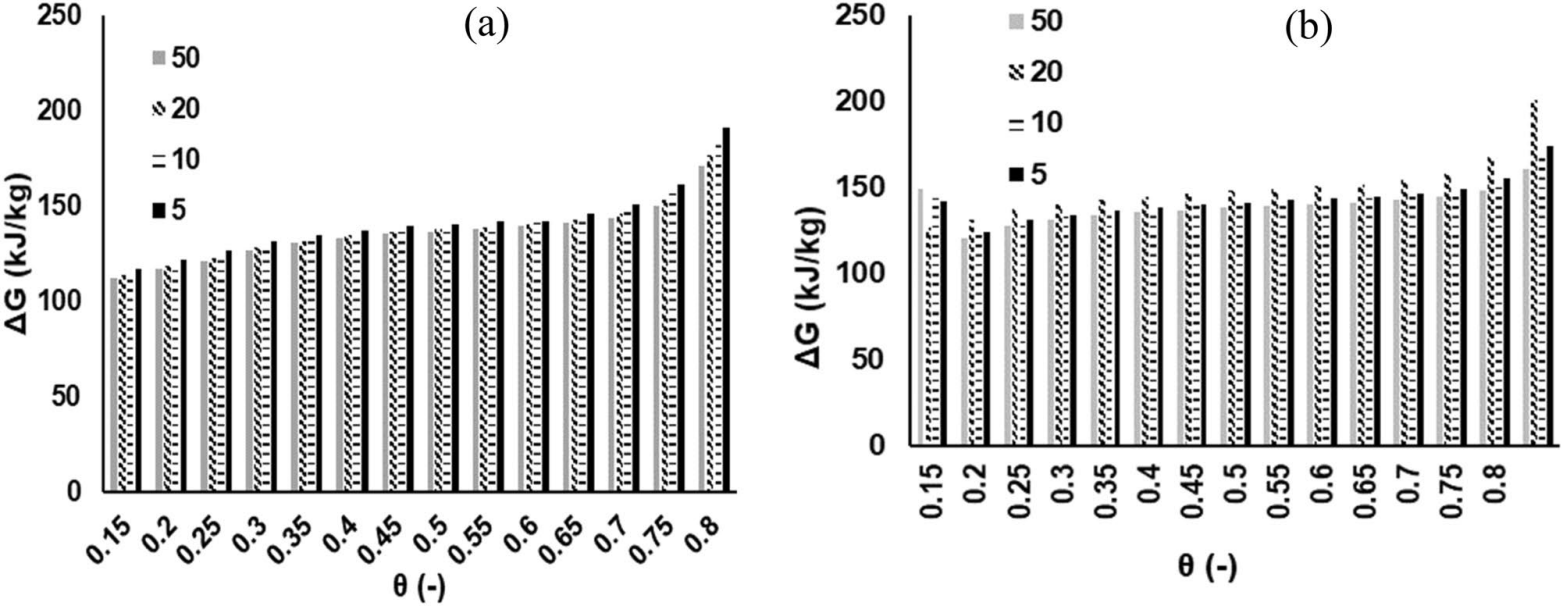
Plot of change of Gibbs free energy ($\Delta G$) at 5, 10, 20, 50 K/min based on DFM (N_2_ pyrolytic environment) for (**a**) MH and (**b**) RP thermal decomposition.

The variations in thermodynamic parameters ($\Delta H$,$~\Delta S$,$\Delta G$) with conversion for MH and RP at multiple heating rates are shown in Figs. 6, 7 and 8 respectively. These data were calculated on the basis of the E_θ_ values derived from DFM, and a 1st order differential model deploying Eqs. (11)–(13). The $\Delta H$ measures the exchange of heat between complex activated constituents and reactants; implying that high values of $\Delta H$ indicate high reactivity and fast reaction rate^33^. It is shown that the average value of $\Delta H$ for the pyrolytic degradation of RP (214 kJ/mol) and MH (233 kJ/mol) are fairly close—suggesting a comparative reactivity as attested to by the DTG profiles in Fig. 1a. A positive value of $\Delta H$ points to an endothermic reaction, while the reverse case is termed an exothermic reaction^26^. It may thus be inferred that for both samples, the pyrolytic decomposition processes encounter endothermic reactions throughout the conversion limit. The difference between the average value of E_θ_ and $\Delta H$ for both samples is approximately 5 kJ/mol—an indication of an ease of product formation from the pyrolytic process^27^. This agrees with findings from literature^34^. The average values of $\Delta H$ obtained in this study are relatively higher in comparison to peanut shell (29 kJ/mol)^27^, and *Typha latifolia* (179 kJ/mol)^34^. The degree of disorder for any reaction during thermal degradation process is determined by the change in entropy, $~\Delta S$. The average value of $~\Delta S$ for MH (0.152 kJ/kg °C) is relatively higher than RP (0.116 kJ/kg °C); and fairly agrees with data in literature^27^. The $\Delta G$ is a measure of the total energy increase during a reaction in a thermal process. It also provides valuable information regarding the spontaneity as well as the direction of the reactions. Chen et al.^26^ noted that a positive value of $\Delta G$ is an indication of non-spontaneity, while a large value suggests that the feasibility of the reaction is low. The mean values of $\Delta G$ for MH and RP pyrolytic degradation processes are 135 and 143 kJ/mol, respectively. The $\Delta G$ data obtained in this study for both samples has the same order of magnitude with those found in previous studies^21,34^. However, they are higher than that for peanut shell and this may indicate a more favorable reaction in relative terms^27^. Additionally, the non-spontaneity associated with the pyrolytic degradation of both MH and RP was visibly demonstrated with the variation in $\Delta G$ showing positive values for all the conversion ratio.

## Conclusion

The study examined the pyrolytic decomposition of two agricultural samples, namely, *M. oleifera* husk and *D. regia* pod in an N_2_ inert atmosphere under dynamic heating conditions. Subsequently, it undertook kinetics modeling based on three isoconversional models, and thermodynamics analyses using non-isothermal thermogravimetric analysis data. A clear distinction was noted in the decomposition profiles of the samples as a more prominent characteristic “shoulder” was displayed by MH. Two distinctive regions were noted from the apparent activation energy profiles with the latter stage presenting higher average E_θ_ values. According to the kinetics analysis the initiation of reactions was relatively easier at the early stages of decomposition. It was also demonstrated that the thermal performance parameters increased with the heating rates. The difference between the average values of activation energy and change in enthalpy was approximately 5 kJ/mol.

## Data Availability

The data will be made available upon request.
